# Anti-inflammatory effects of lavender and eucalyptus essential oils on the in vitro cell culture model of bladder pain syndrome using T24 cells

**DOI:** 10.1186/s12906-022-03604-2

**Published:** 2022-04-30

**Authors:** Adrienn Horváth, Edina Pandur, Katalin Sipos, Giuseppe Micalizzi, Luigi Mondello, Andrea Böszörményi, Péter Birinyi, Györgyi Horváth

**Affiliations:** 1 Department of Pharmacognosy, Faculty of Pharmacy, University of Pécs, H-7624, Rókus u. 2, Pécs, Hungary; 2 Department of Pharmaceutical Biology, Faculty of Pharmacy, University of Pécs, H-7624, Rókus u. 2, Pécs, Hungary; 3 Department of Chemical, Biological, Pharmaceutical and Environmental Sciences, University of Messina, 98168 Messina, Italy; 4 Chromaleont s.r.l., c/o Department of Chemical, Biological, Pharmaceutical and Environmental Sciences, University of Messina, 98168 Messina, Italy; 5 Unit of Food Science and Nutrition, Department of Medicine, University Campus Bio-Medico of Rome, 00128 Rome, Italy; 6 Institute of Pharmacognosy, Faculty of Pharmacy, Semmelweis University, H-1085 Üllői út 26, Budapest, Hungary; 7 Mikszáth Pharmacy, H-1088, Mikszát Kálmán tér 4, Budapest, Hungary

**Keywords:** Bladder pain syndrome, Anti-inflammatory effects, T24 cells, Lavender essential oil, Linalool, eucalyptus essential oil, Eucalyptol

## Abstract

**Background:**

Interstitial cystitis (IC) has a chronic chemical irritation and inflammation of non-bacterial origin in the bladder wall leading to various severe symptoms**.** There is evidence that chronic inflammation is significantly associated with abnormal urothelial barrier function, epithelial dysfunction. This is the underlying cause of urothelial apoptosis and sterile inflammation.

**Method:**

The anti-inflammatory effects of lavender and eucalyptus essential oils (EOs) and their main components (linalool and eucalyptol) were investigated in the T24 human bladder epithelial cell line on TNFα stimulated inflammation, at 3 types of treatment schedule. The mRNA of pro-inflammatory cytokines (IL-1β, IL-6, IL-8) were measured by Real Time PCR. Human IL-8 ELISA measurement was performed as well at 3 types of treatment schedule. The effects of lavender and eucalyptus EOs and their main components were compared to the response to NFκB inhibitor ACHP (2-amino-6-[2-(cyclopropylmethoxy)-6-hydroxyphenyl]-4-(4-piperidinyl)-3-pyridinecarbonitrile).

**Result:**

There is no significant difference statistically, but measurements show that lavender EOs are more effective than eucalyptus EO. Long time treatment (24 h) of both lavender EO and linalool showed higher effect in decreasing pro-inflammatory cytokine mRNA expression than ACHP inhibitor following TNFα pre-treatment. Moreover, both lavender EOs were found to be significantly more effective in decreasing IL-8 secretion of T24 cells after TNFα pre-treatment compared to the ACHP NFκB-inhibitor.

**Conclusion:**

The lavender EOs may be suitable for use as an adjunct to intravesical therapy of IC. Their anti-inflammatory effect could well complement glycosaminoglycan-regenerative therapy in the urinary bladder after appropriate pharmaceutical formulation.

**Supplementary Information:**

The online version contains supplementary material available at 10.1186/s12906-022-03604-2.

## Background

The popularity of essential oils (EOs) has been growing over the last decade. There are EOs such as chamomile, eucalyptus, rosemary, lavender and yarrow that modulate the inflammatory response and are able to influence signalling cascades, regulatory transcription factors, pro-inflammatory gene expression and cytokine production [1]. EOs from lavender and eucalyptus species are widely used in the cosmetic, food and pharmaceutical industries [2] Eucalyptol is the main constituent of eucalyptus oil. Eucalyptol has been shown to inhibit cytokine production and therefore, it is considered to have anti-inflammatory properties [3]. Because of its anti-inflammatory effect, it has been used to treat respiratory diseases, such as bronchitis, sinusitis, bronchial asthma and chronic obstructive pulmonary disease. In addition, its anti-inflammatory effects are exploited even in neurodegenerative diseases, e.g. Alzheimer’s disease [3]. In cardiovascular disease, studies suggest that eucalyptol reduces heart rate via a parasympathetic mechanism and induces hypotension by vasorelaxation. This component also blocks nuclear factor kappa B (NFκB) pathway [4, 5].

The main pharmacologically active constituents of lavender EO are linalool and linalyl acetate. The potent anti-inflammatory effects of both components have been described [6]. Linalool has most commonly been tested in an in vivo LPS-induced lung injury model, cigarette smoke-induced lung inflammation, a carrageenan-induced edema model, and has also been shown to be effective in renal ischaemia [7, 8]. In addition, it is also being tested for adjuvant treatment of various bacterial infections, such as *Pseudomonas aeruginosa* [6, 9]. Linalool has been shown to block the NFκB and MAPK pathways [4].

EOs are complex substances that can be composed of hundreds of components and their composition can vary depending on certain circumstances, such as the origin of the plant, the time of harvest or the extraction method, which may also affect their pharmacological effects [10].

Regulation of the NFκB pathway is very important for cells to adapt to changing environmental conditions. NFκB modulates a wide range of cellular processes such as proliferation, migration, apoptosis, immunity and inflammation. NFκB regulates the pro-inflammatory response by regulating TNFα and interleukin signaling pathways [11]. On the other hand, TNFα is one of the most potent physiological inducers of the nuclear transcription factor NFκB [12]. TNFα, a pro-inflammatory cytokine is involved in a number of regulatory functions, such as cell proliferation, apoptosis and induces the production of other cytokines. TNFα is synthetized primarily by macrophages, but is also expressed in many other cells, including endothelial cells [13].

The bladder pain syndrome (BPS)/ interstitial cystitis (IC) is caused by insufficiency of the mucus layer of the bladder. This could develop into a chronic chemical irritation and inflammation of non-bacterial origin in the bladder wall leading to various severe symptoms e.g. excessive urinary frequency and constant bladder pain [14]. It is estimated that 2.4 to 5.3 million men and 3.5 to 8.6 million women in the United States suffer from symptoms of IC/BPS [15]. It is very difficult to diagnose this syndrome. The main principle of the treatment is to promote the regeneration of the glycosaminoglycan layer with oral and intravesical preparations. The main active components in these preparations are mostly heparin, hyaluronic acid and chondroitin sulfate [16, 17]. IC/BPS is a chronic inflammatory bladder disease. There is evidence that chronic inflammation is significantly associated with abnormal urothelial barrier function, epithelial dysfunction. This is the underlying cause of urothelial apoptosis. It has even been shown to decrease the expression of certain proteins, such as zonula occludens-1 (ZO-1) and E-cadherin [18]. Histopathologically, an infiltration of mast cells in the bladders is observed suggesting that the immune system is involved in the development and maintenance of the disease [19]. For inducing IC/BPS sterile inflammation tumor necrosis factor (TNF) α pro-inflammatory cytokine treatment was used for human bladder epithelial cells [1]. However, TNFα also regulates many cellular functions such as cell proliferation, differentiation, survival and apoptosis. The main producers of TNFα are macrophages, but they are also sensitive to changes in TNFα levels. It also modulates pro-inflammatory cytokine cascade production in macrophages [20]. Correlation was found between IL-1β, IL-6 and IL-8 in the serum of patients and the existence of IC/BPS serum. The increased expression of IL-1β, IL-6, IL-8 in the serum of IC/BPS patients indicates that not only mast cell activation but also some other inflammatory mediators are involved in the pathogenesis of IC/BPS [21, 22].

The inflammatory response of cells eventually results in transcriptional upregulation of TNFα and other pro-inflammatory cytokines e.g. IL-6, IL-1β and IL-8 [10]. The uroepithelial cells secreted IL-8, IL-6 and IL-1β [23] and the blood serum of IC/BPS patients was shown to have increased expression of pro-inflammatory cytokines and chemokines (IL-1β, IL-6, IL-8) [24], therefore we focused on these cytokines.

IL-1β often acts synergistically with TNFα during the pro-inflammatory process, and it induces the production of IL-6 and IL-8, as well. IL-1ß secretion is significantly increased even in autoinflammatory diseases such as chronic infantile neurological, skin and arthritis syndrome, familial cold autoinflammatory syndrome, familial Mediterranean fever [25]. IL-6 plays an important role in inflammation as an inducer of acute phase protein synthesis, and inhibition of TNFα and IL-1β expression by macrophages. IL-6 has simultaneously pro-inflammatory and anti-inflammatory effects [26]. IL-8 acts as a chemokine with chemotactic and activating properties for neutrophils. In addition, IL-8 levels in urine are also used as a marker of disease progression, e.g. in pyelonephritis or idiopathic nephrotic syndrome [27–32].

The aim of our research was to reveal whether lavender and eucalyptus EOs and their main components (linalool and eucalyptol) were able to reduce sterile, chronic inflammation in the bladder at the cellular level during bladder pain syndrome. We examined the extent of anti-inflammatory effect of EOs and the main components by the determination of the changes of pro-inflammatory interleukins (IL-1β; IL-6; IL-8) at mRNA expression level. In addition, we compared the efficacy of EOs with that of the known NFκB-inhibitor ACHP (2-amino-6-[2-(cyclopropylmethoxy)-6-hydroxyphenyl]-4-(4-piperidinyl)-3-pyridinecarbonitrile) to prove the rate of effectiveness of the EOs. Finally, we investigated the anti-inflammatory effects of the components at the secreted protein level of T24 human bladder epithelial cells.

## Materials and methods

### Essential oils

*Lavandula angustifolia* Mill. was collected in two phenophases of the plant: at the beginning of the flowering period (20 June, 2019) and at the end of the flowering period (18 July, 2019). The location of the plant harvesting was: Bolho village (Somogy county, Hungary, coordinates: 46.03904°N 17.30376°E). The EO was extracted from the plant by water-steam distillation based on the description of the 8th edition of the Hungarian Pharmacopoeia (2003). 40 g of dried flowers were used for one distillation. The procedure took 3 h. The EO content was measured with the volumetric method. Three distillation procedures were prepared parallel. The lavender EOs have been examined on THP-1 (human monocyte; Sigma-Aldrich Kft., Budapest, Hungary; 88,081,201) cell line as well [33]. *Eucalytus globulus* Labill. EO was purchased from Aromax (Aromax Zrt., Budapest, Hungary). Linalool and eucalyptol standards were purchased from Sigma-Aldrich (Sigma-Aldrich Kft., Budapest, Hungary). Stock solutions of the essential oils were prepared by adding 10% (22 μg/mL) (100 μL) dimethyl sulfoxide (100% DMSO, Sigma-Aldrich Ltd., Budapest, Hungary) to 900 μL of essential oil. The emulsions were vortexed and diluted 500-fold with phosphate buffered saline (PBS, Lonza Ltd., Basel, Switzerland). Stock solutions of linalool and eucalyptol standards were prepared in the same way as EOs.

### GC-MS and GC-FID analysis

Analyzes were carried out according to our previous study [33]. Briefly, distilled EOs were solubilized in methanol solvent (dil. 1:100) and injected into GC instrumentations for the chromatographic separation. In this respect, the identification of the volatile components was carried out by using a gas chromatograph GCMS-QP2020 (Shimadzu, Duisburg, Germany) equipped with a single quadrupole mass spectrometer. An AOC-20i auto-sampler was used for the injection of distilled oils. Injection volume was 0.5 μL with a split ratio (split/splitless injector) of 1:10. The capillary column was a low-polarity one, namely SLB-5 ms 30 m × 0.25 mm ID, 0.25 μm d_*f*_ (Merck Life Science). The separation of components was performed in temperature gradient as follows: from 50 °C to 300 °C at 3.0 °C/min. Helium was used as carrier gas, at an initial inlet pressure of 26.7 kPa and at an average linear velocity of 30 cm/s. MS parameters were as follows: mass range: 40–550 amu; ion source temperature: 220 °C; interface temperature: 250 °C. GCMS solution software (version 4.52, Shimadzu) was used for data collection and data processing. As detailly described in Micalizzi et al. [34], two different identification parameters were utilized for peak assignment: spectral similarity (over 85%) and a ± 5 LRI tolerance window. FFNSC mass spectral library version 4.0 (Chromaleont, Messina, Italy) was mainly used for the identification of analytes. Quantitative analyses were carried out by using a gas chromatograph GC-2010 (Shimadzu) equipped with a flame ionization detector (GC-FID). Capillary column, temperature gradient and gas carrier were the same used in GC-MS analyses except for initial inlet pressure (99.5 kPa). FID parameters were as follows: temperature: 300 °C; hydrogen flow: 40 mL/min; air flow: 400 mL/min; nitrogen flow (make-up gas): 30 mL/min; sampling rate: 40 ms. LabSolutions software (version 5.92, Shimadzu) was used for the acquisition of chromatograms and their processing [35].

### Cell culture and treatments

T24 human bladder epithelial cells (HTB-4; American Type Culture Collection, USA) were maintained in McCoy’s 5A Medium Modified (Lonza, Ltd., Basel, Switzerland) supplemented with 10% fetal bovine serum (FBS, EuroClone S.p.A, Pero, Italy) and 1% penicillin/streptomycin (Lonza Ltd., Basel, Switzerland).

T24 cells were seeded in 6-well plates and cultured for 24 h before treatments. The inflammation was induced using TNFα treatment (10 ng/mL, Shenandoah Biotechnology Inc. Warminster PA, USA) for 6 h and 24 h. TNFα concentration was determined by concentration dependence experiments followed by the analysis of pro-inflammatory cytokine mRNA expression (Supplementary Fig. 2.) The anti-inflammatory effects of EOs and their main components were determined in three different experiments: TNFα pre-treatment for 6 h followed by 24 h of EO/standard treatment; TNFα pre-treatment for 24 h followed by 6 h of EO/standard treatment; and TNFα pre-treatment for 24 h followed by 24 h of EO/standard treatment. The EOs and standards were used at 500-fold dilution of stock solutions to determine their effect on cytokine production. In all experiments, cells treated with DMSO were used as controls. All experiments were performed at least 3 times and carried out at 37 °C in a humidified atmosphere containing 5% CO_2_.

### Cell viability assay

T24 cells were plated onto 96-well plates using 5000 cells/well. The cells were treated with EOs and standards in 200-fold (0.16 μg/mL), 500-fold (0.1 μg/mL), 1000-fold (0.05 μg/mL) and 2000-fold (0.025 μg/mL) dilutions for 6 h and 24 h. Viability of the T24 cells were measured using Cell Counting Kit-8 (CCK-8) (Sigma-Aldrich Kft., Budapest, Hungary) after the treatments. Cells treated with DMSO were used as a control for cells treated with EOs, while the effect of DMSO on cell viability was used as a control for untreated cells. A DMSO stock solution was also prepared (10 μL of 100% DMSO was diluted with 990 μL of phosphate buffered saline (PBS, Lonza Ltd., Basel, Switzerland) and further dilutions (200-fold (0.16 μg/mL), 500-fold (0,1 μg/mL), 1000-fold (0.05 μg/mL), and 2000-fold (0.025 μg/mL) were performed. After treatment, 10 μL of WST-8 reagent was applied to each well and all plates were incubated for 1 h at 37 °C and 5% CO_2_. After the incubation time, 10 μL of 1% sodium dodecyl sulfate (SDS, Molar Chemicals Ltd., Halásztelek, Hungary) was pipetted to each well to stop the reaction. Then, the absorbance of the samples was determined at 450 nm with a MultiSkan GO microplate spectrophotometer (Thermo Fisher Scientific Inc., Waltham, MA). This workflow can be found in Supplementary Fig. 3. Cell viability was expressed as a percentage of the total cell number of the corresponding control. The effect of TNFα treatment on cell viability was also determined using different concentrations (1 ng/mL, 5 ng/mL; 10 ng/mL) [36]. These results can be found in Supplementary Table 2.

### Real-time PCR

T24 cells were treated as previously described in 6-well culture dishes (4 × 10^5^ cells/well). After treatments, T24 cells were rinsed with PBS, and finally, after trypsinization, the cell pellets were collected. Then, samples were subjected to total RNA isolation using the Quick RNA mini kit (Zymo Research, Irvine, CA). The complementary DNA was synthesized from 200 ng of total RNA following the protocol of the high capacity cDNA Reverse Transcription Kit (Applied Biosystems, Thermo Fisher Scientific Inc., Waltham, MA). Gene expression was measured in 20 μL total reaction volume using a CFX96 real-time PCR system (Bio-Rad Inc., Hercules, CA) with iTaq™ Universal SYBR® Green Supermix (Bio-Rad Inc., Hercules, CA). After running quantitative PCR, melting curves were generated to confirm that a single specific product was recovered. The Livak (2-∆∆Ct) method was used for relative quantification using a Bio-Rad CFX Maestro software (Bio-Rad Inc., Hercules, CA). The workflow can be found in Supplementary Fig. 4. The gene expression levels obtained in the samples were plotted against β-actin levels. Thus, relative expression levels were obtained and used to compare untreated and treated samples. The relative expression level of the controls was regarded as 1., then the mRNA expression of treated cells was determined relative to controls [37]. The primer sequences used in this study are described in Table 1.

**Table 1:** Real-time PCR gene primer list

Primer	Sequence 5′ → 3’
IL-6 forward	CTGAGAAAGGAGACATGTAACAAG
IL-6 reverse	GGCAAGTCTCCTCATTGAATC
IL-8 forward	CAGTGCATAAAGACATACTCC
IL-8 reverse	CACTCTCAATCACTCTCAGT
IL-1β forward	GAAATGATGGCTTATTACAGTGG
IL-1β reverse	GGTGGTCGGAGATTCGTA
TNFα forward	CTCTCTCTAATCAGCCCTCT
TNFα reverse	CTTGAGGGTTTGCTACAACA
β-actin forward	AGAAAATCTGGCACCACACC
β-actin reverse	GGGGTGTTGAAGGTCTCAAA

### Enzyme-linked immunosorbent assay (ELISA) measurements

After each treatment of cells, the culture supernatant of control and treated cells was collected and kept at − 80 °C until measurements. The concentration of IL-8 secreted in the culture media was determined using human IL-8 ELISA kit (Thermo Fisher Scientific Inc., Waltham, MA) following the manufacturer’s instructions [38].

### Statistical analysis

Statistical analysis was performed using SPSS software (IBM Corporation, Armonk, NY, USA). Statistical significance was determined by one-way ANOVA followed by Tukey’s HSD post hoc test. Data are shown as mean ± standard deviation (SD). Statistical significance was set at *p* value < 0.05. Each assay was carried out in triplicate in each independent experiments (*n* = 3).

## Results

### Effect of essential oils and standards on T24 human bladder epithelial cell viability

**Fig. 1:**
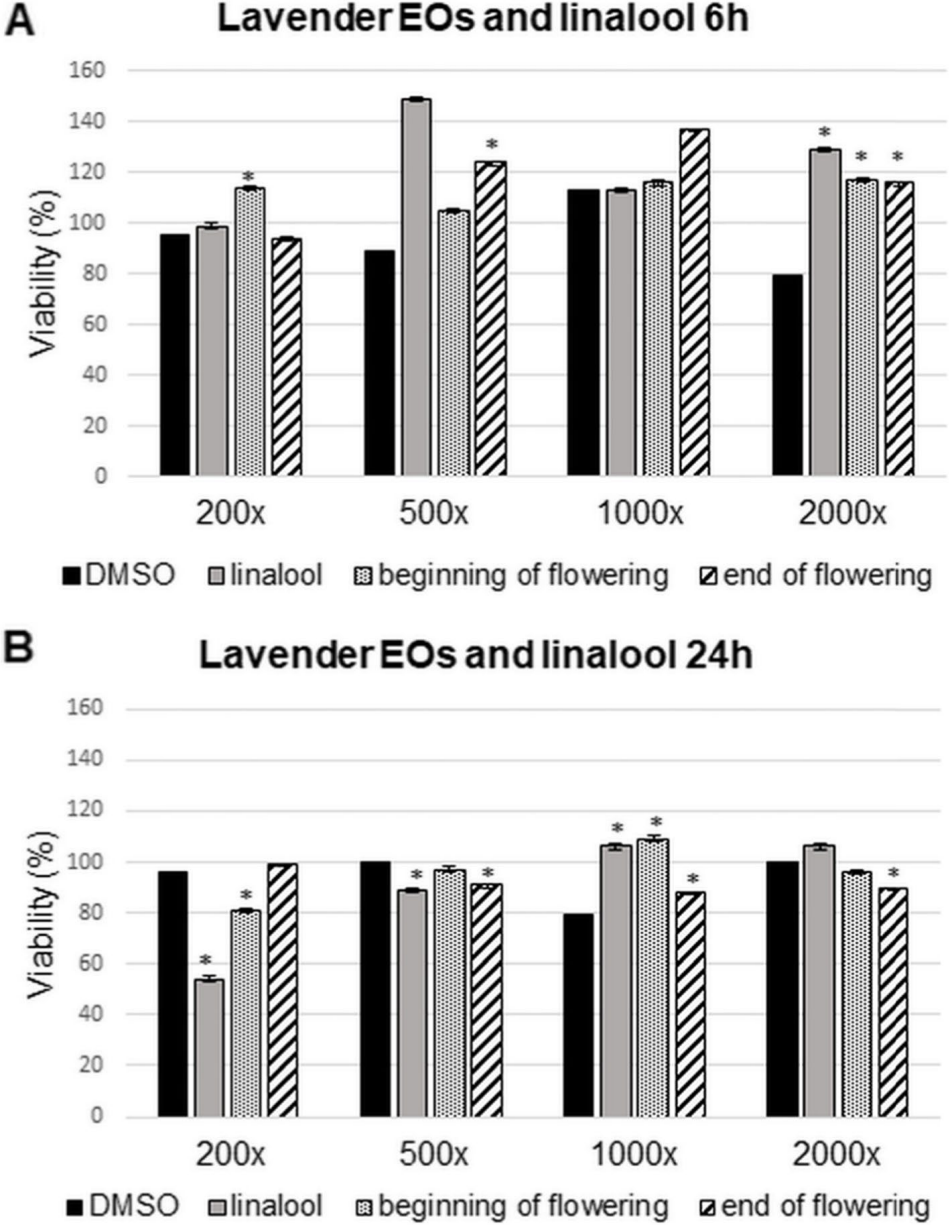
Cell viability assays of lavender EOs and linalool on T24 cells. Viability of the cells were determined using CCK-8 kit. **A** Cells were treated by linalool and lavender EOs prepared at the beginning of flowering and the end of flowering period with four different dilutions, for 6 h. **B** Cells were treated by linalool and lavender EOs prepared at the beginning of flowering and the end of flowering period using four different dilutions, for 24 h. Control cells were treated with the same dilutions of DMSO for 6 h or 24 h. The experiments were repeated three (*n* = 3) times in triplicates. Asterisks indicate *p* < 0.05 compared to the controls

**Fig. 2:**
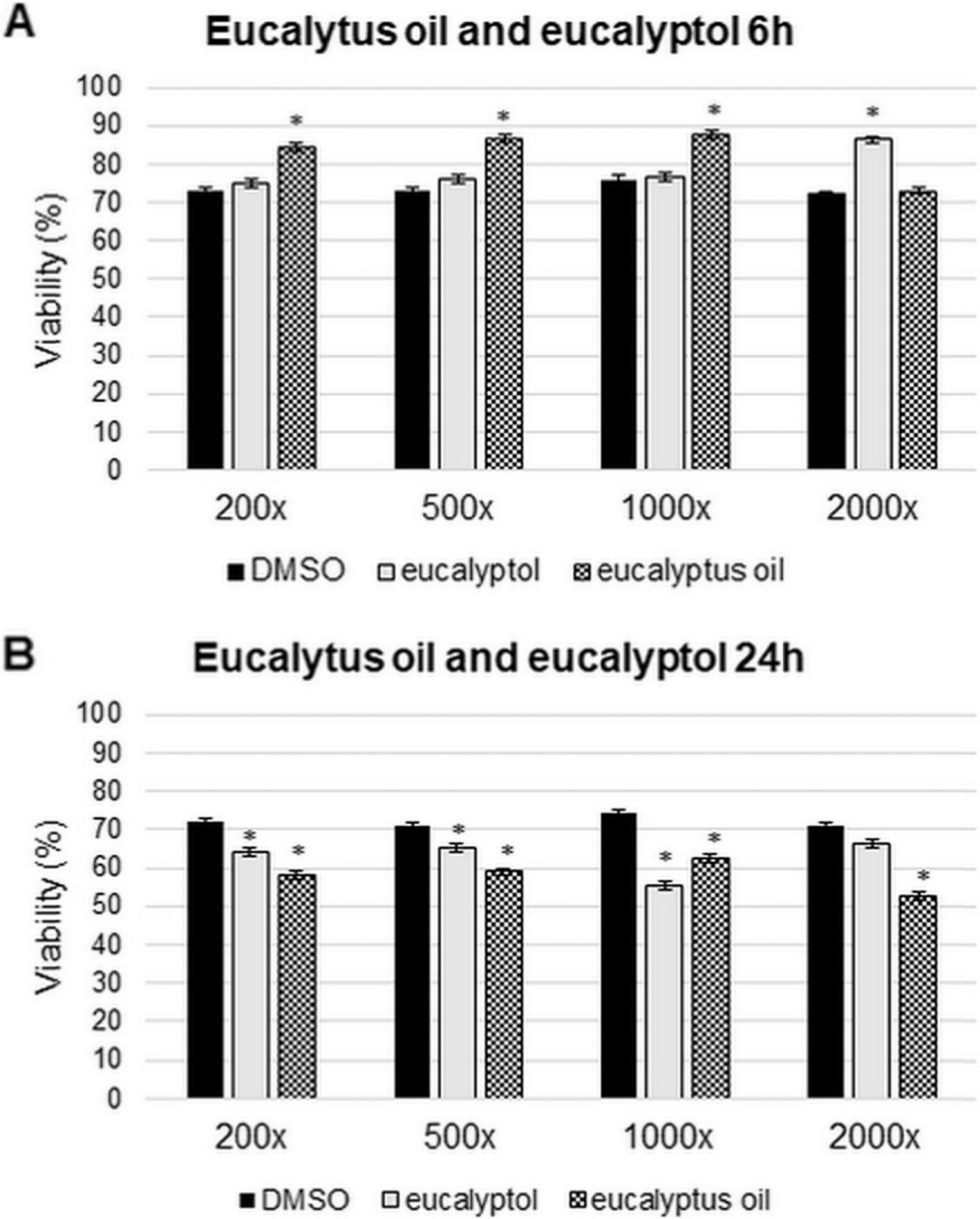
Cell viability assays of eucalyptus oil and eucalyptol on T24. Cell viability was determined using CCK-8 kit. **A** Cells were treated by eucalyptol and eucalyptus oil with four different dilutions, for 6 h. **B** Cells were treated by eucalyptol and eucalyptus oil with four different dilutions, for 24 h. Control cells were treated with the same dilutions of DMSO for 6 h or 24 h. The experiments were repeated three times (*n* = 3) in triplicates. Asterisks indicate *p* < 0.05 compared to the controls

To determine the appropriate dilution of the EOs and standards used in our experiments, first we carried out viability assays on T24 human bladder epithelial cells, because too high concentrations of the components may cause cell death [1, 34]. Viability tests were carried out after 6 h and 24 h treatments with four dilutions (200-fold (0.16 μg/mL), 500-fold (0.1 μg/mL) and 1000-fold (0.05 μg/mL) to 2000-fold (0.025 μg/mL) of the EOs. DMSO being the vehicle of the stock solutions was also tested. Lavender oils and linalool at 6 h treatment did not induce a decrease in cell viability (Fig. 1A). In the 24 h treatments, at 200-fold dilution, linalool was found to be highly toxic to cells, while lavender oil prepared at the beginning of flowering period was only slightly toxic (Fig. 1B). At 500-fold dilutions, cell viability was no longer significantly affected by either linalool or lavender oils at either 6 h or 24 h. At 1000-fold and 2000-fold dilutions neither linalool standard nor lavender oils were toxic at either duration of treatments (Figure 1A and B). In the case of eucalyptus oil and eucalyptol, none of the substances tested were toxic to cells in any of the dilutions after 6 h of treatment (Fig. 2A). Toxicity could be detected in 24 h studies. Figure 2B shows that the 500-fold (0.1 μg/mL) dilution was the least harmful to the cells. Considering the above results we decided to use the 500-fold (0.1 μg/mL) dilutions of all materials. A correlation between viability and EO composition can be assumed (Supplementary Table 1. and Supplementary Fig. 1.).

### Effects of lavender essential oils and linalool standard on mRNA expression of IL-1β, IL-6 and IL-8 after TNFα pre-treatment of T24 cells

As IC disease is characterized by non-bacterial inflammation of the bladder, TNFα was used as a pre-treatment to induce inflammation to mimic the clinical symptom. The uroepithelial cells secret IL-8, IL-6 and IL-1β, therefore we focused on these cytokines. T24 cells were treated in different schedule with linalool and lavender EOs for 6 h and 24 h after TNFα pre-treatment (6 h or 24 h durations) to find out if they have beneficial effects on mRNA levels of the above mentioned cytokines. The appropriate dilution of DMSO was administered to the control cells.

**Fig. 3:**
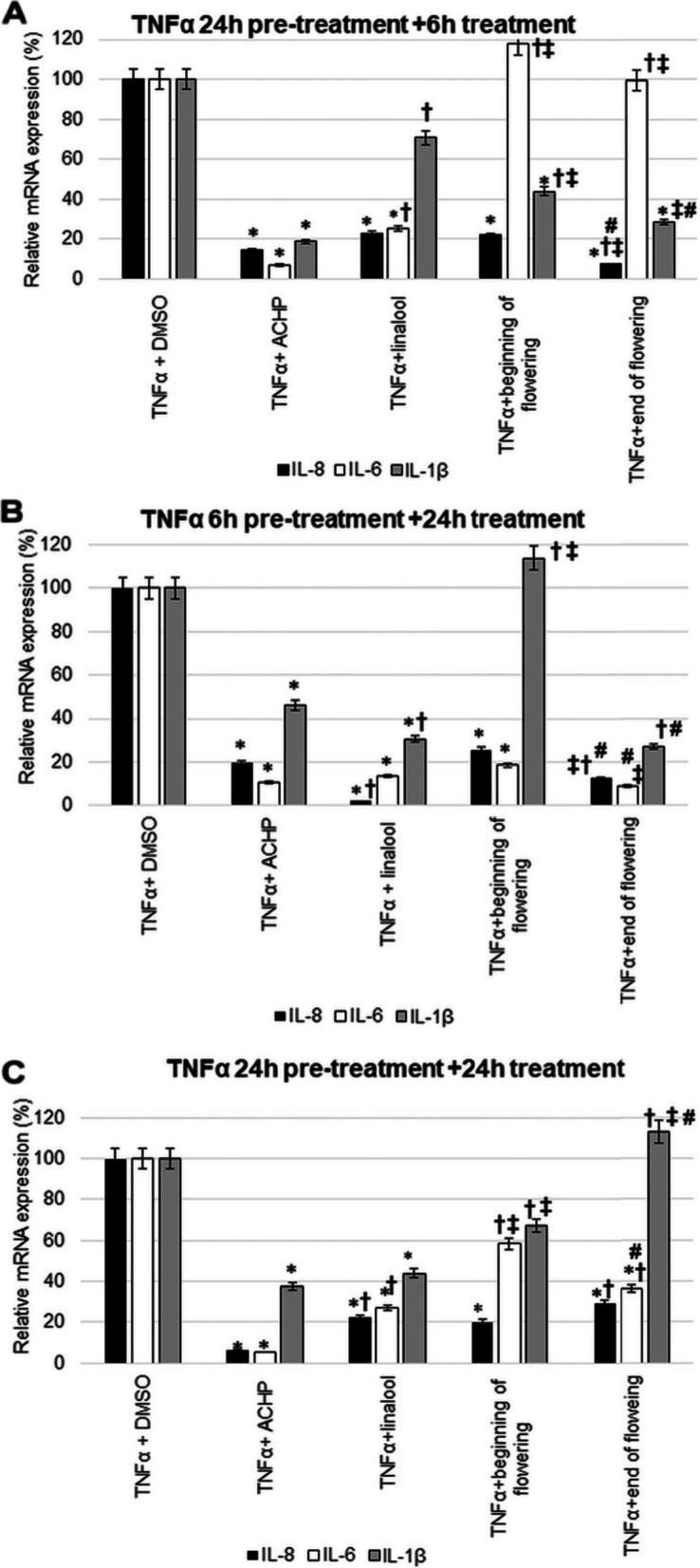
Relative mRNA expression of IL-1β, IL-6 and IL-8 after TNFα pre-treatment of T24 and comparison of the effects of lavender oils and linalool standard with ACHP treatment. The mRNA expression levels were determined with Real-time PCR using SYBR Green protocol. The relative expression of controls was regarded as 1. β-actin was utilized for normalization. **A** T24 cells were pre-treated with TNFα for 24 h then with ACHP, linalool and lavender EOs beginning of flowering and end of flowering for 6 h. **B** T24 cells were pre-treated with TNFα for 6 h then with ACHP, linalool and lavender EOs beginning of flowering and end of flowering for 24 h. **C** T24 cells were pre-treated with TNFα for 24 h then with ACHP, linalool and lavender EOs beginning of flowering and end of flowering for 24 h. DMSO treatment was used as a control of the treated cells. The experiments were repeated three times (*n* = 3) in triplicates*.* Asterisks indicate *p* < 0.05 compared to the control. Cross marks *p* < 0.05 compared to the ACHP treatment. Double cross shows *p* < 0.05 compared to the linalool treatment. Number sign indicates *p* < 0.05 compared to the beginning of flowering

A significant reduction in IL-8 mRNA expression was observed for all oils and standard treatments (Fig. 3). Some treatments were very outstanding. In case of IL-8, using 6 h EO treatment after 24 h of TNFα pre-treatment, EO distilled from lavender at the end of flowering period was found to be the most effective. The change of IL-6 was only considered to be effective the linalool standard. For the short treatment (6 h) after TNFα 24 h pre-treatment, the highest IL-1β expression alteration was measured for the EO obtained from the end of flowering (Fig. 3A).

In case of IL-6 mRNA levels, the most effective treatment was 24 h EO treatment after 6 h of TNFα pre-treatment. Based on changes in IL-8 the linalool standard proved to be more effective than EOs. A decrease in IL-1β mRNA expression was observed 24 h after 6 h TNFα pre-treatment with linalool standard and end of flowering EO (24 h) (Fig. 3B).

For the long treatment (24 h) after TNFα 24 h pre-treatment, the greatest change in mRNA expression was observed for IL-8. In this case, the most effective was the beginning of flowering lavender EO. In the case of IL-6 again, the effect was most pronounced for linalool. The greatest reduction in IL-1β expression was seen with the linalool standard. Based on the IL-1β mRNA expression, this treatment was proved to be the least effective in this case (Fig. 3C).

As lavender EO exert their anti-inflammatory effects via the NFκB pathway, the activity of lavender EOs and linalool was compared to the efficacy of ACHP treatment that inhibits the entire NFκB pathway. Compared to the DMSO control, the ACHP inhibitor significantly reduced the expression of all examined cytokines (Fig. 3).

After TNFα 24 h pre-treatment, IL-6 and IL-8 mRNA expressions decreased most after 6 h of ACHP treatment. It should be highlighted that lavender EO prepared at the end of flowering showed better result than the ACHP inhibitor for IL-8 (Fig. 3A).

The 24 h ACHP treatment following 6 h TNFα pretreatment significantly reduced IL-6, IL-8 and IL-1β expression (Fig. 3B). Compared to the ACHP treatment, better results for IL-8 were obtained with linalool and EO from end of flowering period. Moreover, for IL-6, EO from end of flowering was more effective than ACHP treatment. In case of IL-1β, the alteration in mRNA expression in response to linalool treatment and end of flowering EO was more beneficial than the ACHP NFκB inhibitor treatment (Fig. 3B).

At 24 h TNFα pre-treatment and 24 h ACHP treatment, both IL-6 and IL-8 expressions were reduced in almost equal extent. EOs and linalool were not effective compared to ACHP treatment in this case (Fig. 3C).

### Effects of eucalyptus essential oil and eucalyptol standard on mRNA expression of IL-1β, IL-6 and IL-8 after TNFα pre-treatment

Similarly to lavender EOs and linalool experiments, sterile inflammation was induced in T24 cells by TNFα. The effects of eucalyptus oil and eucalyptol standard were also investigated by monitoring the changes in IL-1β, IL-6 and IL-8 expression.

**Fig. 4:**
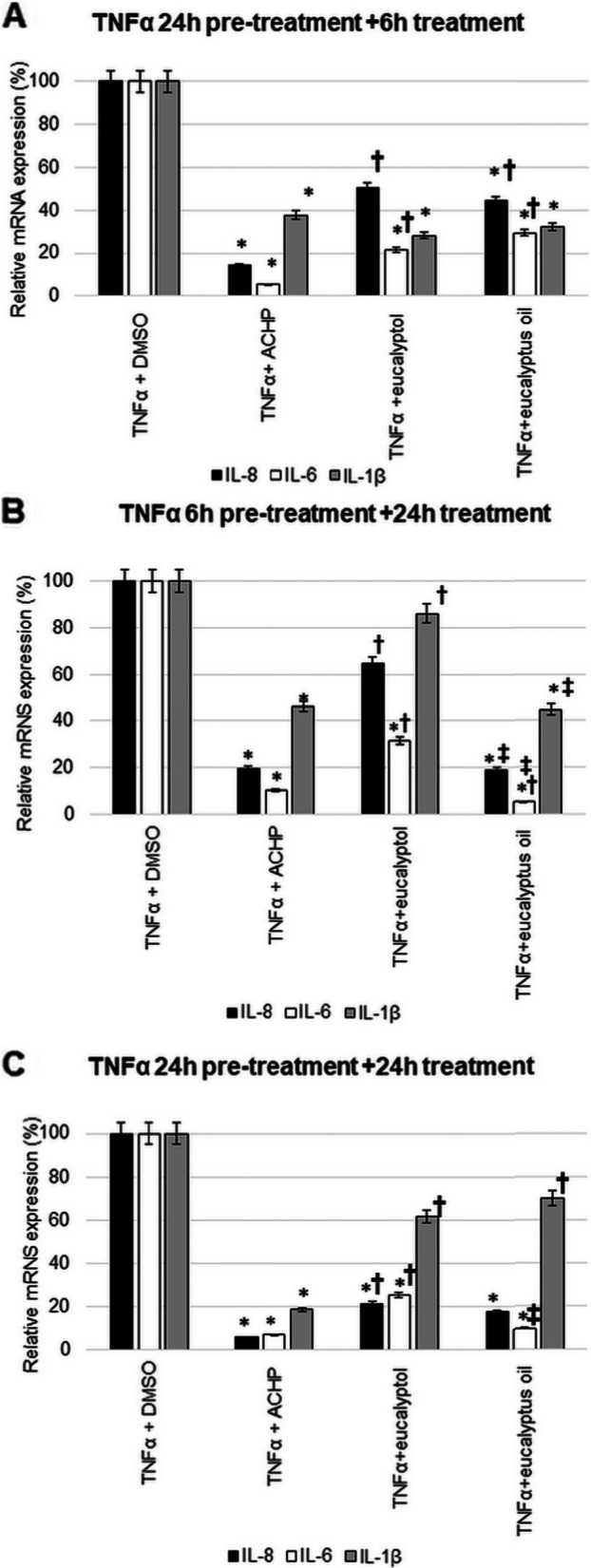
Relative mRNA expression of IL-1β, IL-6 and IL-8 after TNFα pre-treatment of T24 and comparison of standard effects of eucalyptol and eucalyptus oils with ACHP treatment. The mRNA expression levels were determined with Real-time PCR using SYBR Green protocol. The relative expression of controls was regarded as 1. β-actin was utilized for normalization. **A** T24 cells were pre-treated with TNFα for 24 h then 6 h treated with ACHP/eucalyptol and eucalyptus oil. **B** T24 cells were pre-treated with TNFα for 6 h then 24 h treated with ACHP/ eucalyptol and eucalyptus oil **(C)** T24 cells were pre-treated with TNFα for 24 h then 24 h treated with ACHP/ eucalyptol and eucalyptus oil. DMSO treatment was used as a control of the treated cells. The experiments were repeated three times (*n* = 3) in triplicates*.* Asterisks indicate *p* < 0.05 compared to the control. Cross marks *p* < 0.05 compared to the ACHP treatment. Double cross shows *p* < 0.05 compared to the eucalyptol treatment

Comparing the effects of eucalyptus EO and eucalyptol in the case of IL-8 expression, after 24 h of TNFα pre-treatment, the eucalyptus EO was better for 6 h of treatments. IL-1β and IL-6, mRNA expression was effectively reduced by both eucalyptus oil and eucalyptol. There was no significant difference in results between the two substances (Fig. 4A).

The alteration in IL-8 chemokine expression after 6 h of TNFα pre-treatment was found to be more promising for 24 h of treatments with eucalyptus oil. The largest significant reduction in IL-6 mRNA expression changes was observed after 6 h of TNFα pretreatment with long treatment (24 h). For IL-1β, a significant reduction was seen again with eucalyptus EO treatment (Fig. 4B).

The IL-8 and IL-6 mRNA expression in case of 24 h TNFα pre-treatment following 24 h treatment with eucalyptol standard and eucalyptus EO was decrease significantly. Comparing these two results, the eucalyptus EO was more effective. No significant change was observed in IL-1β with this treatment protocol (Fig. 4C).

The effect of eucalyptus EO and eucalyptol standard on the inhibition of NFκB pathway was also compared to the ACHP treatment.

After 24 h of TNFα pre-treatment and 6 h of ACHP treatment, the decrease of IL-6 expression was the most significant (Fig. 4A). Neither eucalyptus oil nor eucalyptol was more effective than the inhibitor treatment for any of the cytokines (Fig. 4A).

Again, the mRNA expression levels obtained at 24 h of ACHP treatment after 6 h of TNFα pre-treatment showed the largest decrease for IL-6, followed by IL-8 and IL-1β. More effective treatment than ACHP was seen for both IL-8 and IL-6 with the eucalyptol EO (Fig. 4B).

### IL-8 enzyme-linked immunosorbent assay (ELISA) measurement at T24 cells with linalool, lavender oils, eucalyptol and eucalyptus oil treatments after TNFα pre-treatment

The most promising inhibitory effect of the examined EOs and their main compounds was found in case of IL-8 chemokine. Therefore, secreted IL-8 protein concentrations were also determined. Significant reductions were measured 24 h after short term TNFα pre-treatment and 6 h after long term TNFα pre-treatment in response to ACHP inhibitor treatment.

**Fig. 5:**
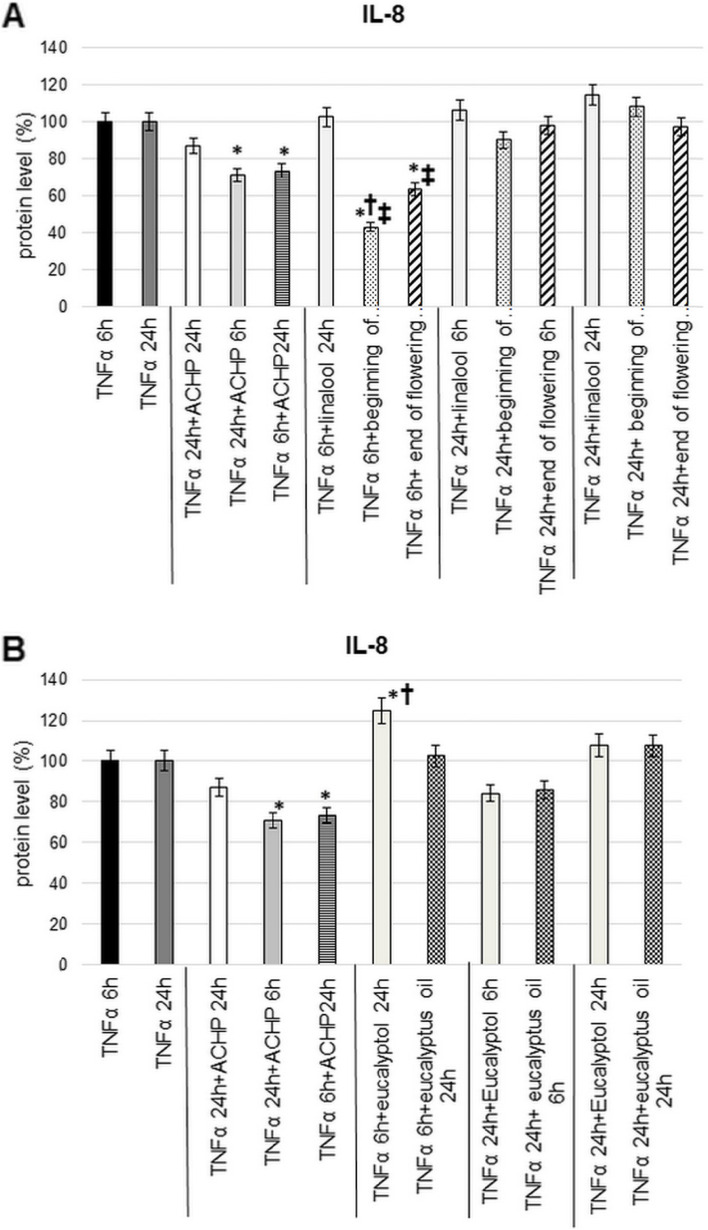
Human IL-8 ELISA measurement at 3 types of treatment schedule. **A** Secreted IL-8 protein level determination of TNFα pre-treated T24 cells after treated with ACHP, linalool and lavender EOs: beginning of flowering and end of flowering. **B** Secreted IL-8 protein level determination of TNFα pre-treated T24 cells after treated with ACHP, eucalyptol, and eucalyptus oil. The experiments were repeated three times (*n* = 3) in triplicates. Asterisks indicate *p* < 0.05 compared to the TNFα pre-treatments. Cross marks *p* < 0.05 compared to the ACHP treatment*.* Double cross shows *p* < 0.05 compared to the linalool treatment

Lavender EOs significantly reduced IL-8 protein levels 24 h after 6 h of TNFα pre-treatment. Moreover, the EO distilled from the beginning of flowering was more effective (Fig. 5A). No change was observed for linalool. Treatments with EO of lavender or linalool standard after 24 h TNFα pre-treatment were not capable to decrease the IL-8 protein levels (Fig. 5A).

In the case of eucalyptus EO and eucalyptol standard treatments, there was no change in EO after 6 h of TNFα pre-treatment and 24 h of treatment, whereas eucalyptol significantly increased IL-8 level (Fig. 5B). After 24 h of TNFα pre-treatment, 6 h of eucalyptus oil and eucalyptol treatments caused a decrease, while 24 h of treatment caused increase in IL-8 levels (Fig. 5B).

## Discussion

**Fig. 6:**
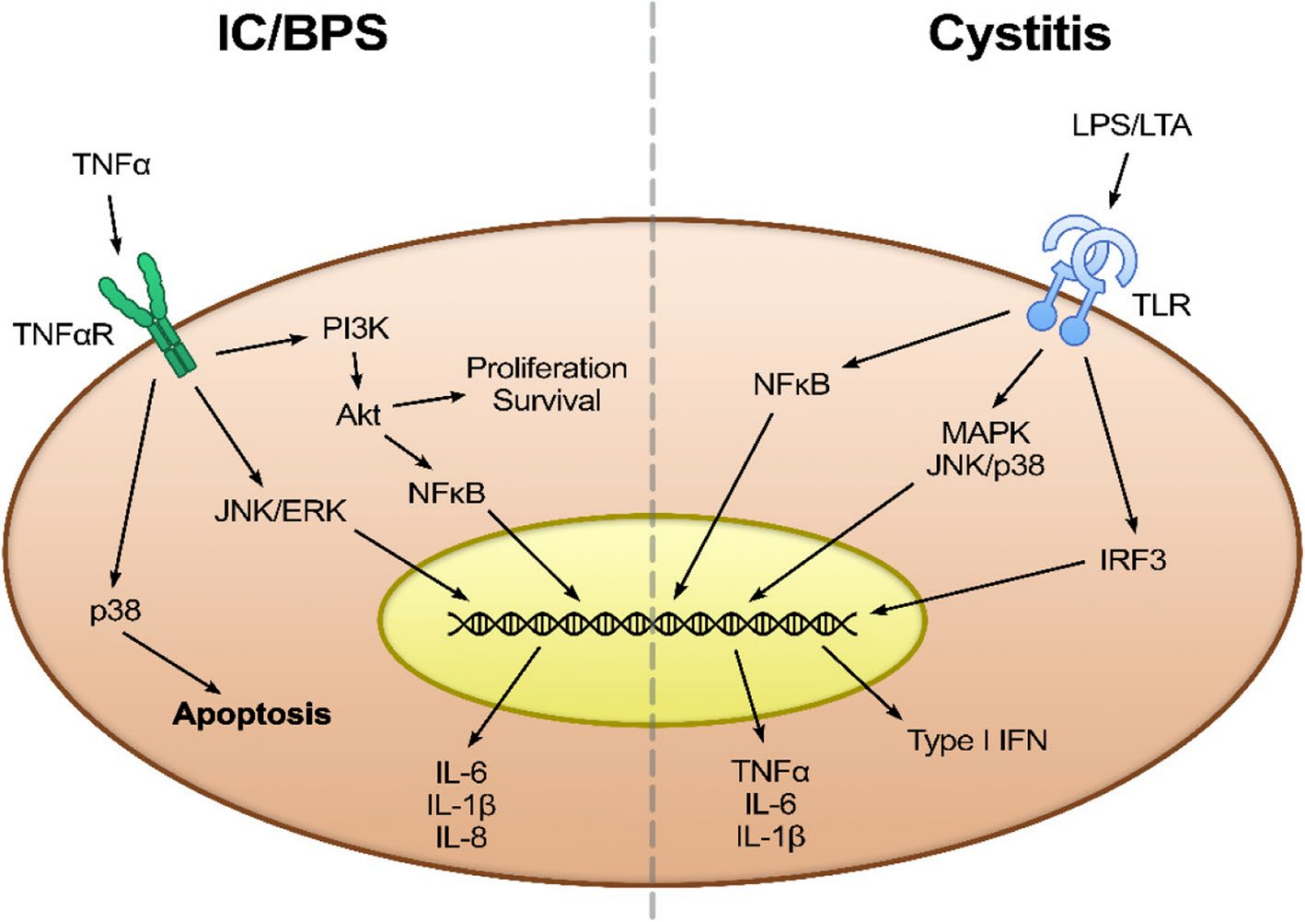
Comparison of IC/BPS signaling pathway and cystitis signaling pathway

In this study, we examined the effects of lavender and eucalyptus EOs and their main components (linalool and eucalyptol) in an in vitro T24 cell culture (human bladder epithelial cells) modelling of IC/BPS. It is important to highlight that the IC/BPS is a non-bacterial inflammation of the bladder wall, only the symptoms are similar to bacterial cystitis. Usually the cystitis is caused by Gram negative bacterial infection and the signaling pathway starts with activation by lipopolysaccharide (LPS) that is the common virulence factor [39]. The LPS targets the Toll-like receptors (TLRs) [40]. The TLRs are involved in the expression of transcriptional mediators, which leads to the production of pro-inflammatory cytokines and Type I IFNs, such as IL-1β, IL-6, IL-8, INF-γ, and TNF-α [41, 42]. In contrast, sterile inflammation of IC/BPS increases TNFα activity, which acts through TNFα receptors, similarly leading to pro-inflammatory interleukin production (Fig. 6).

Utilization of EOs against bacterial infections is emerging due to the increased occurrence of antibiotic resistance. Hybrid lavender EOs seem to be promising candidates as new therapies against the multidrug-resistant *Pseudomonas aeruginosa* due to their low cytotoxicity and high effectiveness [38]. Lavender oils from true lavender, spike lavender and hybrid lavenders differ in the constitution and therefore in biological and therapeutic activities. Lavender EOs are also potent candidates as anti-cancer therapy since they affect cell morphology, cell cycle and proliferation of cancer cells [34]. Further examination of the lavender EOs may lead to the development of innovative therapies and could be used for new clinical applications.

Our studies showed that the most pronounced reduction in the cytokine mRNA expression in response to lavender oils and linalool treatments was obtained in all cases after short time (6 h) TNFα pre-treatment. Furthermore, linalool and lavender EO prepared at the end of flowering period were the most effective. Similar results could be observed when comparison was made with ACHP NFκB inhibitor treatment, which was utilized as a positive control of cytokine transcription inhibition. For both IL-8 and IL-1β, lavender EO prepared at the end of flowering and linalool had better effect than the ACHP treatment. Treatment with end of flowering lavender EO for 6 h after long TNFα pre-treatment showed better efficacy on IL-8 cytokine expression compared to the ACHP treatment. The lavender oil distilled at end of flowering was presumably more effective than EO produced at beginning of flowering (36.33 ± 0.10%) because of its linalool content was higher (41.63 ± 0.13%). When comparing the composition of lavender EOs based on GC-MS, the differences are clearly observed. It contains the second highest percentage of linalyl acetate and terpinene-4-ol, after linalool. The major components of the beginning of flowering EO are linalool (36.33%; 632 ng/mL), linalyl acetate (21.47%; 386.46 ng/mL) and terpinene-4-ol (9.47%; 176.71 ng/mL). Meanwhile, end of flowering lavender EO contains higher level of linalool (41.63%; 724 ng/mL) and terpinene-4-ol (16.69%; 311.43 ng/mL), and lower level of linalyl acetate (14.78%; 266.04 ng/mL) (Supplementary Table 1). All these active substances have a proven anti-inflammatory effect and inhibit the NFκB pathway [8, 20, 43, 44]. α-Terpineol may also contribute to the anti-inflammatory effect of the EOs since in vivo it reduces the serum levels of pro-inflammatory cytokines, such as IL-1β, IL-6 and TNF-α [33, 45, 46].

Comparisons between eucalyptus oil and eucalyptol in terms of effect on mRNA expression showed overall higher potency of the EO for all cytokines. Furthermore, compared to the effect of ACHP NFκB inhibitor, long treatments with eucalyptus EO were more effective for IL-8 and IL-6. The results revealed that the eucalyptus EO was more effective than the main component eucalyptol. The complex effect of EO causes more decrease in mRNA expression. In addition to eucalyptol, α-pinene, γ-terpinene, terpinen-4-ol and α-terpineol may contribute to the anti-inflammatory effect of eucalyptus EO [46, 47]. The inhibitory effects of α-pinene on inflammatory responses are significantly decreased the production of the IL-6 and TNFα [48]. γ-terpinene decreases the production of pro-inflammatory cytokines, such as IL-1β and IL-6, and enhance IL-10 [49]. Examining IL-8 chemokine at protein level confirms that the examined EOs and components were the most effective in case of short induction of sterile inflammation. EOs distilled from lavender at the beginning of flowering and at the end of flowering significantly reduced IL-8 levels and even proved to be more effective than ACHP treatment.

Our studies clearly demonstrate that the components of the EO are crucial in determining the anti-inflammatory effect. Lavender EOs were found to be more effective than eucalyptus oil in reducing inflammation. The reason for this, based on GC, can be traced back to the components. When comparing the two main components analysed, it is evident from the results that linalool has a stronger inhibitory effect than eucalyptol. Lavender EOs also contain high levels (min 4%) of linalyl acetate, terpinen-4-ol, α-terpineol. Eucalyptus oil, on the other hand, contains only small amounts of other components besides eucalyptol, which could complement its effect, such as α-pinene (6.32%; 108.45 ng/mL), γ-terpinen (3.2%; 54.40 ng/mL) and terpinen-4-ol (1.9%; 35.36 ng/mL). The overlap between the constitution of the two essential oils is minimal and the amounts are small (0.02–1.6%). The common components are eucalyptol, terpinen-4-ol, α-terpineol, mycrene, α-pinene, β-pinen, γ-terpinen, α-phellandrene. In some cases the effect of these small percentages of components may be negligible (Supplementary Table 1).

Based on our study the lavender EOs are more effectively reduce the inflammatory components in this in vitro system than eucalyptus EO. Both lavender EOs and linalool were more effective in decreasing pro-inflammatory cytokine mRNA expression than ACHP inhibitor, suggesting that the different components of EO affect not only the NFκB pathway, but others (e.g. MAPK), as well. Moreover, both lavender EOs were found to be significant effective in short TNF pre-treatment after long EO treatment, compared to the ACHP NFκB-inhibitor in the IL-8 ELISA assay. However, the beginning of flowering lavender EO was better than end of flowering EO. Part of the reason for this may be that linalyl acetate has a delayed onset of action compared to linalool, due to in vivo biotransformation, which increases linalool concentration in the EO [50]. On the other side, there may be translation factors involved in the process that influence its effect. Additional experiments are needed for revealing these effects. In summary, we may say that the different EOs have distinct therapeutic effects at certain inflammatory conditions, as our study demonstrates. Even the timing of the harvesting of flowers may have importance because of the composition of the EO. The composition of EOs can also vary from year to year due to weather conditions and different extraction techniques, which should always be considered when experimenting with them. Examination of EOs obtained from different years may result in the determination of the best ratio of the components, which gives the possibility for the development of “artificial” EOs with high efficiency.

## Conclusions

In our study, we investigated the anti-inflammatory effects of lavender and eucalyptus EOs and their main components on T24 human uroepithelial cell line. We mimicked the sterile inflammation of the IC/BPS induced by TNFα, which was the pre-treatment. In conclusion, our results showed that lavender EOs were more effective than eucalyptus EO in this case. Furthermore, we concluded that, comparing the protein levels of IL-8 pro-inflammatory cytokine secreted by the T24 cells treated with EOs and standards, with the inhibitory effect of ACHP inhibitor, the lavender EOs showed better efficacy than ACHP treatment. Comparing lavender EOs with each other, lavender EO prepared at the beginning of flowering period was the more effective. This is presumably due to the different composition of the two EOs.

Finally, lavender EOs may be suitable for use as an adjunct to intravesical therapy. Their anti-inflammatory effect may well complement GAG-regenerative therapy in the bladder after pharmaceutical formulation.

## Supplementary Information

**Additional file 1: Supplementary Table 1.** Percentage (%) and relative concentrations (ng/mL) of the compounds of eucalyptus essential oil; *LRI _exp_: experimental linear retention index. *The components structure come from PubChem. *RT: Retention time. **Supplementary Table 2.** Cell viability assays of TNFα on T24. **Supplementary Fig. 1.** Eucalyptus oil chromatogram. **Supplementary Fig. 2.** mRNA expression of TNFα for 6 h and 24 h treatments. **Supplementary Fig. 3.** Cell viability workflow. **Supplementary Fig. 4.** Real time PCR workflow

## Data Availability

All data generated or analysed during this study are included in this published article. The datasets generated and/or analysed during the current study are available in the Mendeley Data repository, doi: 10.17632/6m9xsj5t35.1. https://data.mendeley.com/datasets/6m9xsj5t35/

## Abbreviations

ACHP: 2-Amino-6-[2-(cyclopropylmethoxy)-6-hydroxyphenyl]-4-(4-piperidinyl)-3-pyridinecarbonitrile
BPS: Bladder Pain Syndrome
CCK-8: Cell counting kit-8
DMSO: Dimethyl sulphoxide
ELISA: Enzyme-linked immunosorbent assay
EOs: Essential oils
FBS: Fetal bovine serum
GC-FID: Gas chromatography flame ionisation detector
GC-MS: Gas chromatography mass spectrometer
IC: Interstitial cystitis
IL-1β: Interleukin-1β
IL-6: Interleukin-6
IL-8: Interleukin-8
LPS: Lipopolysaccharide
MAPK: Mitogen activated protein kinase
NFκB: Nuclear factor kappaB
PBS: Phosphate buffered saline
TNFα: Tumor necrosis factor α
T24: Human bladder epithelial cells line
