# Supplementary material for: Anti-inflammatory effects of lavender and eucalyptus essential oils on the in vitro cell culture model of bladder pain syndrome using T24 cells
Source: BMC Complement Med Ther. 2022 Apr 30;22:119. doi: 10.1186/s12906-022-03604-2 (PMC9055718; doi:10.1186/s12906-022-03604-2)

Anti-inflammatory effects of lavender and eucalyptus essential oils on the *in vitro* cell culture model of bladder pain syndrome using T24 cells

Adrienn Horváth [1,2], Edina Pandur[2], Katalin Sipos [2], Giuseppe Micalizzi[3], Luigi Mondello [3,4,5],Andrea Böszörményi[6], Péter Birinyi[7] and Györgyi Horváth[1,*]

Supplementary figure 4. Real time PCR workflow

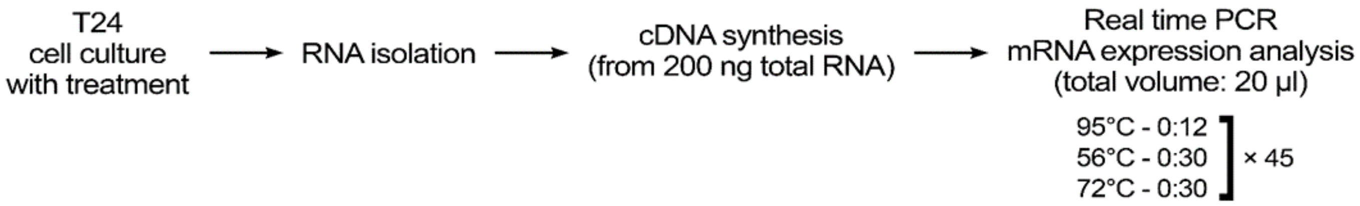

Supplement: Supplementary file 1 — Additional file 1: Supplementary Table 1. Percentage (%) and relative concentrations (ng/mL) of the compounds of eucalyptus essential oil; *LRI exp: experimental linear retention index. *The components structure come from PubChem. *RT: Retention time. Supplementary Table 2. Cell viability assays of TNFα on T24. Supplementary Fig. 1. Eucalyptus oil chromatogram. Supplementary Fig. 2. mRNA expression of TNFα for 6 h and 24 h treatments. Supplementary Fig. 3. Cell viability workflow. Supplementary Fig. 4. Real time PCR workflow [file 12906_2022_3604_MOESM1_ESM.zip › Supplementary figure 4_.pdf]
