# Supplementary material for: Anti-inflammatory effects of lavender and eucalyptus essential oils on the in vitro cell culture model of bladder pain syndrome using T24 cells
Source: BMC Complement Med Ther. 2022 Apr 30;22:119. doi: 10.1186/s12906-022-03604-2 (PMC9055718; doi:10.1186/s12906-022-03604-2)
Supplement: Supplementary file 1 — Additional file 1: Supplementary Table 1. Percentage (%) and relative concentrations (ng/mL) of the compounds of eucalyptus essential oil; *LRI exp: experimental linear retention index. *The components structure come from PubChem. *RT: Retention time. Supplementary Table 2. Cell viability assays of TNFα on T24. Supplementary Fig. 1. Eucalyptus oil chromatogram. Supplementary Fig. 2. mRNA expression of TNFα for 6 h and 24 h treatments. Supplementary Fig. 3. Cell viability workflow. Supplementary Fig. 4. Real time PCR workflow [file 12906_2022_3604_MOESM1_ESM.zip › Supplementary table 1_.pdf]

Anti-inflammatory effects of lavender and eucalyptus essential oils on the *in vitro* cell culture model of bladder pain syndrome using T24 cells

Adrienn Horváth [1,2], Edina Pandur[2], Katalin Sipos [2], Giuseppe Micalizzi[3], Luigi Mondello [3,4,5],Andrea Böszörményi[6], Péter Birinyi[7] and Györgyi Horváth[1,*]

Supplementary table 1. Percentage (%) and relative concentrations (ng/mL) of the compounds of eucalyptus essential oil

Compounds	Structure	LRI_{exp}	RT (min)	Percentage of compound in the essential oils[a]	Relative concentration of compound in the experiments (ng/mL)[b]
				eucalyptus EO	eucalyptus EO
α-Pinene CID 11240513)	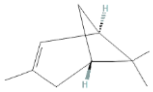	933	5.1	6.32	108.45
β-Pinene (CID 14896)	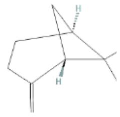	977	6.1	0.70	12.03
Myrcene (CID 31253)	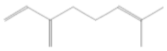	988	6.4	1.00	15.82
α-Phellandrene (CID: 443160)	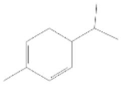	1006	6.7	1.60	27.20
α-Terpinene (CID: 7462)	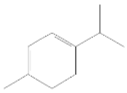	1017	7	0.50	8.37
Eucalyptol (CID: 2758)	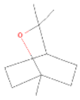	1032	7.5	82.3	1515.96

γ-Terpinene (CID: 7461)	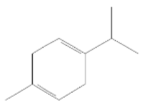	1058	7.9	3.20	54.40
Terpinen-4-ol (CID: 5325830)	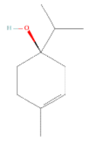	1182	10.6	1.90	35.38
α-Terpineol (CID: 17100)	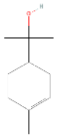	1197	10.9	1.50	27.90
β-Terpineol (CID: 17100)	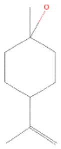	1147	10.3	0.6	11.00
**Total**				99.50	

*LRI $_{exp}$: experimental linear retention index

*The components structure come from PubChem

*RT: Retention time
